# Testosterone ameliorates vascular aging via the Gas6/Axl signaling pathway

**DOI:** 10.18632/aging.103584

**Published:** 2020-07-27

**Authors:** Yan-Qing Chen, Hui-Min Zhou, Fang-Fang Chen, Ya-Peng Liu, Lu Han, Ming Song, Zhi-Hao Wang, Wei Zhang, Yuan-Yuan Shang, Ming Zhong

**Affiliations:** 1 The Key Laboratory of Cardiovascular Remodeling and Function Research, Chinese Ministry of Education, Chinese National Health Commission and Chinese Academy of Medical Sciences, The State and Shandong Province Joint Key Laboratory of Translational Cardiovascular Medicine, Department of Cardiology, Qilu Hospital of Shandong University, Jinan, Shandong, China; 2 Department of Geriatrics, The Second Hospital of Shandong University, Jinan, Shandong, China; 3 Department of General Practice, Qilu Hospital of Shandong University, Jinan, Shandong, China; 4 Department of Geriatric Medicine, Qilu Hospital of Shandong University, Key Laboratory of Cardiovascular Proteomics of Shandong Province, Jinan, Shandong, China

**Keywords:** vascular aging, testosterone, Gas6, Axl, vascular stiffening

## Abstract

Low serum testosterone level is associated with aging-related vascular stiffness, but the underlying mechanism is unclear. The Growth arrest-specific protein 6 (Gas6) /Axl pathway has been proved to play important roles in cell senescence. In this study, we intend to explore whether Gas6/Axl is involved in the effect of testosterone on vascular aging amelioration. Vascular aging models of wild type and Axl^-/-^ mice were established by natural aging. Mice of these two gene types were randomized into young group, aging group and testosterone undecanoate (TU) treatment group. Mice were treated with TU (37.9 mg/kg) in the TU group, which treated with solvent reagent served as control. The aging mice exhibited decreases in serum testosterone, Gas6 and Axl levels and an increase in cell senescence, manifested age-related vascular remodeling. Testosterone treatment induced testosterone and Gas6 levels in serum, and ameliorated cell senescence and vascular remodeling in aging mice. Furthermore, we uncover the underlying molecular mechanism and show that testosterone treatment restored the phosphorylation of Akt and FoxO1a. Axl knockout accelerated cell senescence and vascular remodeling, and resisted the anti-aging effect of testosterone. Testosterone might exert a protective effect on vascular aging by improving cell senescence and vascular remodeling through the Gas6/Axl pathway.

## INTRODUCTION

Increasing age is not only accompanied by senility, but also accompanied by the age-related diseases, such as atherosclerosis, hypertension and other cardiovascular diseases. In recent years, the aging population has turned gradually obvious, the incidence and mortality of age-related cardiovascular diseases are at a high level. Therefore, there is an urgent need to explore the mechanism of vascular aging, which could provide a basis for clinical intervention. Vascular aging is a major independent risk factor and fertile ground for cardiovascular diseases, such as hypertension, and atherosclerosis [1–3]. The aging vessels exhibit a series of characteristics such as intima-media thickening, luminal dilation, elastic membrane fragmentation, vascular stiffness, endothelial dysfunction and raised blood pressure [4]. Testosterone, the most important male hormone, decreases gradually with age [5]. Testosterone deficiency has emerged as an important predictor of future cardiovascular events and all-cause mortality, and was associated with the markers of vascular aging such as increased carotid intima-media thickness and aortic calcification [6, 7]. Testosterone levels were independently associated with aortic stiffness, and the effect of low testosterone concentration on aortic stiffness was more prominent in young men [8]. Cellular senescence was considered to be involved in the pathogenesis and development of vascular aging [9, 10], and it has been shown that the anti-senescence effect of testosterone is dependent on preventing cell senescence. Testosterone could prevent endothelial senescence and neuronal senescence in SAMP8 mice and cardiomyocytes senescence [11]. However, the mechanism of testosterone in delaying vascular aging and cellular senescence remains to be elucidated.

Growth arrest-specific protein 6 (Gas6), a member of the vitamin K-dependent protein family, interacts with receptor tyrosine kinases of the TAM (Tyro-3, Axl, Mer) family via its C-terminal sex hormone binding globulin (SHBG)-like domain, and is involved in many pathophysiological processes of cardiovascular system [12]. Gas6 has the highest affinity for Axl among TAM receptors, and it is often called Gas6/Axl pathway [13]. Vascular endothelial cells, vascular smooth muscle cells (VSMCs), and fibroblasts can synthesize and express Gas6 and Axl. Recent studies have indicated that Gas6/Axl participated in the development of vascular calcification, vascular remodeling and atherosclerosis [14–19]. In patients with cardiovascular heart disease, serum Gas6 and testosterone levels were much lower, and serum Gas6 concentrations were positively associated with testosterone concentrations [20]. Otherwise, Gas6 concentrations were inversely associated with ages [21]. Testosterone might directly activate androgen response elements of the Gas6 gene to influence Gas6 gene expression and protein production [22]. In our previous study, we found that testosterone could promote the expression of Gas6 in VSMCs, and the Gas6/Axl pathway was involved in the anti-senescence effect of testosterone on angiotensin II-induced VSMCs senescence [23].

Therefore, we hypothesize that testosterone can delay vascular aging, and the Gas6/Axl pathway plays an essential role in the progression of cell senescence and age-related vascular remodeling. In this study, we used wild type (WT) and Axl^-/-^ mice to establish a vascular aging model by natural aging, and then treated with testosterone to explore the mechanism of vascular aging. Firstly, we tested changes of cell senescence, vascular stiffness, blood pressure, and MMPs in aging vessel, and the improving effect of testosterone on the above changes. Then we discussed the influence of Axl knockout on the improving effect of testosterone on vascular aging. Finally, we explored the protective molecular mechanism of the Gas6/Axl pathway in vascular aging.

## RESULTS

### TU treatment up-regulated testosterone and Gas6/Axl expression in aging mice

**Figure 1:**
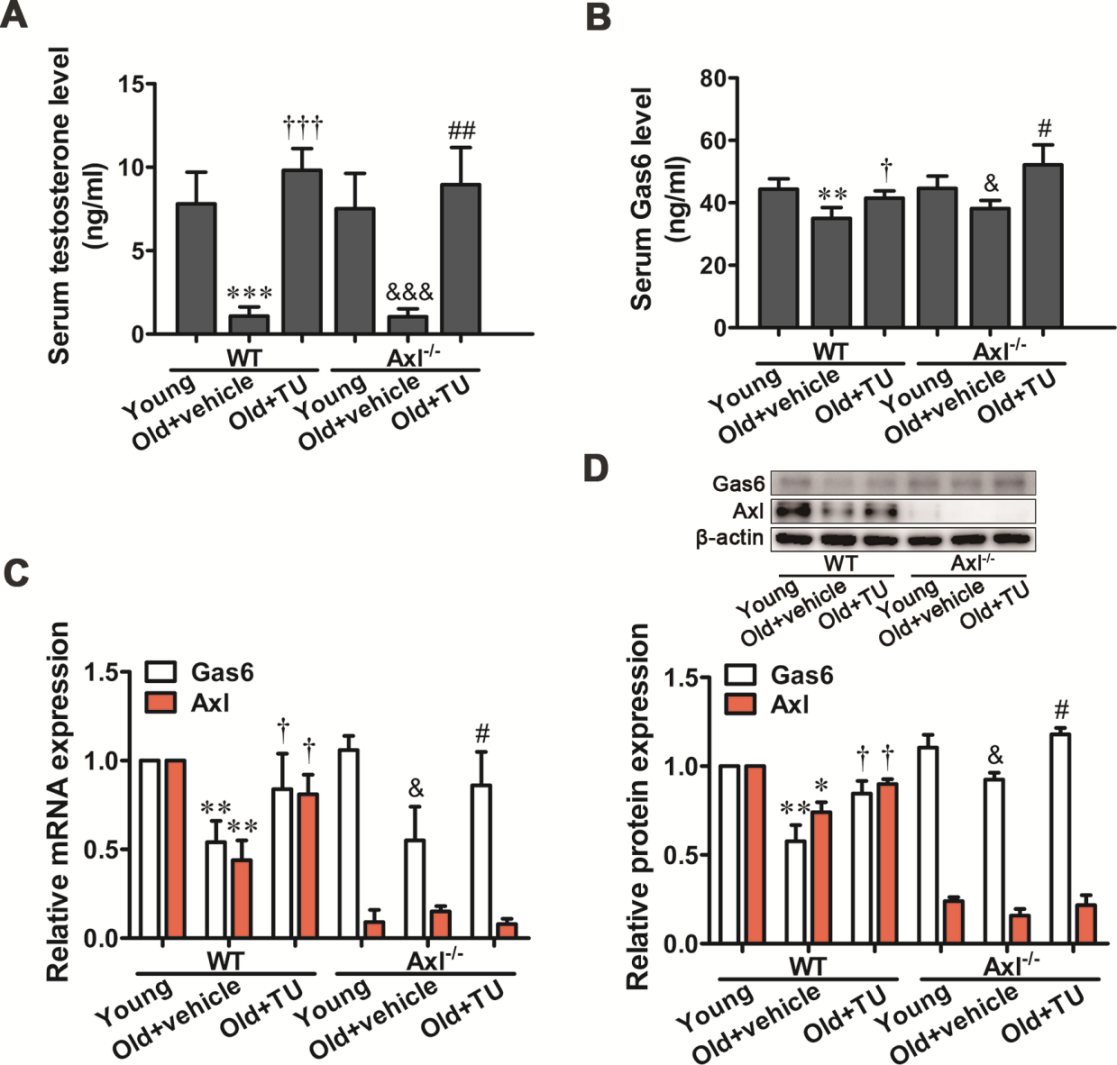
**TU treatment up-regulated testosterone and Gas6/Axl expression in aging mice.** (**A**) The serum level of testosterone. (**B**) The serum level of Gas6. (**C**) Relative mRNA expression of Gas6 and Axl. (**D**) Relative protein content of Gas6 and Axl. Data are mean ± SD; ^*^P < 0.05, ^**^P < 0.01 and ^***^P < 0.001 *vs.* WT young group; ^†^*P* < 0.05 and ^‡^*P* < 0.001 *vs.* WT old group; ^&^*P* < 0.05 and ^&&&^*P* < 0.001 *vs.* Axl^-/-^ young group; ^#^*P* < 0.05 and ^##^*P* < 0.01 *vs.* Axl^-/-^ old group; A-B: n = 8 for each group, C-D: n = 3 for each group.

Serum testosterone level was significantly decreased in both WT (*P*<0.001) and Axl^-/-^ (*P*<0.001) old groups compared with WT and Axl^-/-^ young groups (Figure 1A). With testosterone undecanoate (TU) treatment, the serum testosterone level in WT (*P*<0.001) and Axl^-/-^ (*P*<0.01) old+TU groups was increased compared with WT and Axl^-/-^ old groups (Figure 1A).

The serum Gas6 content was markedly decreased in WT and Axl^-/-^ old groups (*P*<0.05) compared with WT and Axl^-/-^ young groups (Figure 1B). TU replacement therapy significantly increased the serum Gas6 level in WT and Axl^-/-^ old+TU groups compared with WT and Axl^-/-^ old groups (*P*<0.05) (Figure 1B). Similarly, the mRNA and protein expression of Gas6 in aorta were markedly decreased in WT and Axl^-/-^ old groups (*P*<0.05~*P*<0.01) compared with WT and Axl^-/-^ young groups (Figure 1C, 1D). With TU replacement therapy, the mRNA and protein expression of Gas6 in aorta were increased in WT and Axl^-/-^ old+TU groups compared with WT and Axl^-/-^ old groups (*P*<0.05) (Figure 1C, 1D).

The mRNA and protein expression of Axl were decreased in WT old group compared with WT young group (*P*<0.05~*P*<0.01) (Figure 1C, 1D). With TU treatment, the Axl mRNA and protein expression levels were elevated in WT old+TU group compared with WT old group (*P*<0.05) (Figure 1C, 1D). Both mRNA and protein expression of Axl were quite low in all Axl knockout mice (Figure 1C, 1D).

### Gas6/Axl was involved in the anti-senescence effect of testosterone on vascular cell senescence

**Figure 2:**
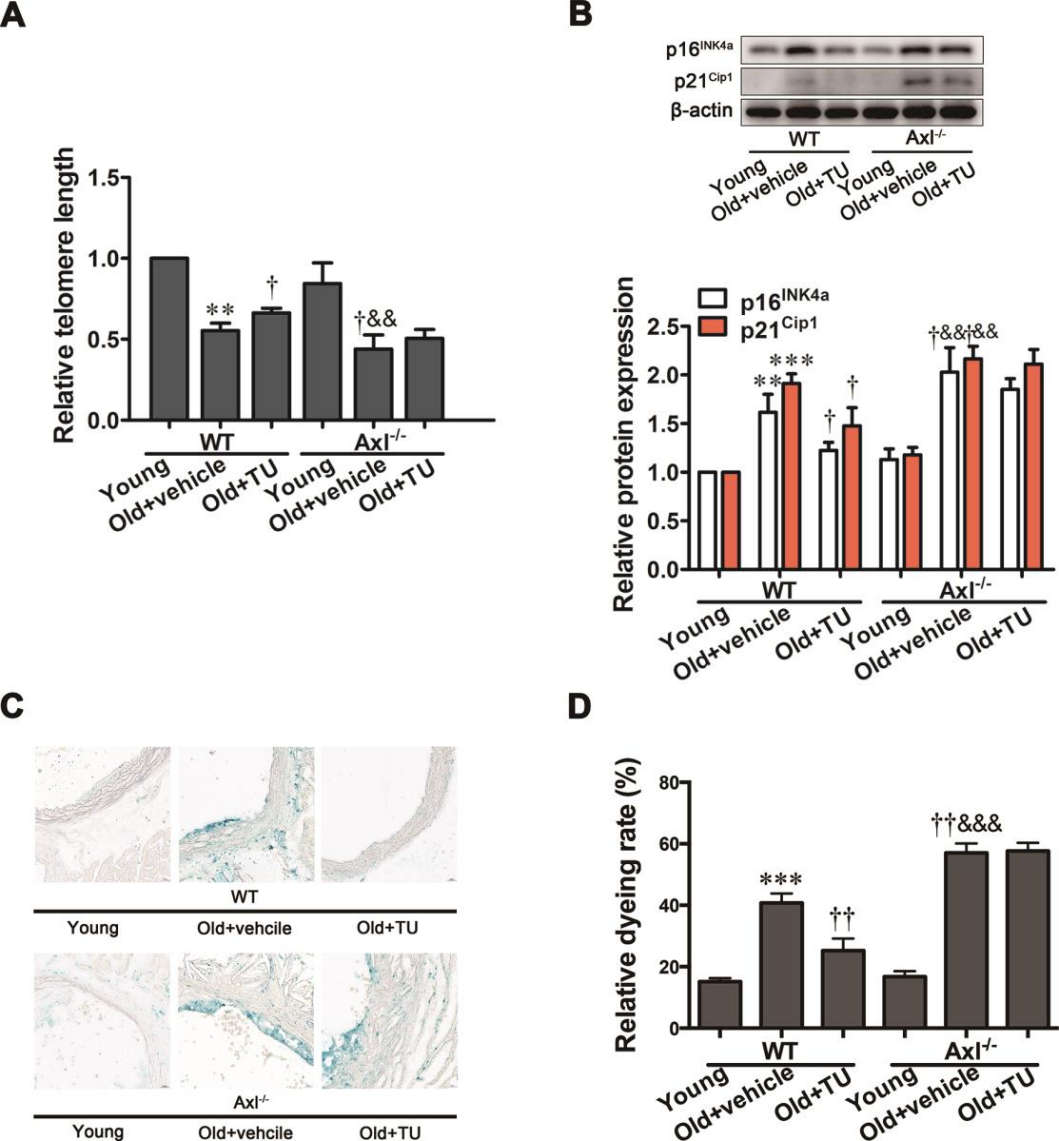
**Gas6/Axl was involved in the anti-senescence effect of testosterone on vascular cell senescence.** (**A**) The relative telomere length was detected by real-time PCR. (**B**) Representative Western blot and analysis of p16^INK4a^ and p21^Cip1^. (**C**): Representative b-galactosidase staining with the descending aorta from the mouse (scale bar: 20μm). (**D**): Analysis of the relative dyeing rate. Data are mean ± SD; ^**^P < 0.01 and ^***^P < 0.001 *vs.* WT young group; ^†^*P* < 0.05 and ^††^*P* < 0.01 *vs.* WT old group; ^&&^*P* < 0.01 and ^&&&^*P* < 0.001 *vs.* Axl^-/-^ young group; A-B: n = 3 for each group, C-D: n = 5 for each group.

Telomere length was significantly shortened in WT (*P*<0.01) and Axl^-/-^ (*P*<0.01) old groups compared to WT and Axl^-/-^ young groups (Figure 2A), and telomere length in Axl^-/-^ old group was shorter than WT old group (*P*<0.05) (Figure 2A). With TU treatment, the telomere length in WT old+TU group was significantly longer than WT old group (*P*<0.05) (Figure 2A). Telomere length in Axl^-/-^ old+TU group was not longer than Axl^-/-^ old group (Figure 2A). The protein expression of p16^INK4a^ and p21^Cip1^ were increased in WT and Axl^-/-^ old groups compared with WT and Axl^-/-^ young groups (*P*<0.01~*P*<0.001) (Figure 2B). The Axl^-/-^ old group had the highest p16^INK4a^ (*P*<0.05) and p21^Cip1^ (*P*<0.05) expression compared with all other groups (Figure 2B). With TU treatment, the expression of p16^INK4a^ (*P*<0.05) and p21^Cip1^ (*P*<0.05) in WT old+TU group was significantly decreased compared with WT old group (Figure 2B). But the expression of p16^INK4a^ and p21^Cip1^ in Axl^-/-^ old+TU group were not significantly decreased compared with Axl^-/-^ old group (Figure 2B). The changing trend of SA-β-Gal staining rate was the same as that of p16^INK4a^ and p21^Cip1^ (Figure 2C, 2D).

### The role of testosterone and Gas6/Axl in structural changes of aging vessel

**Figure 3:**
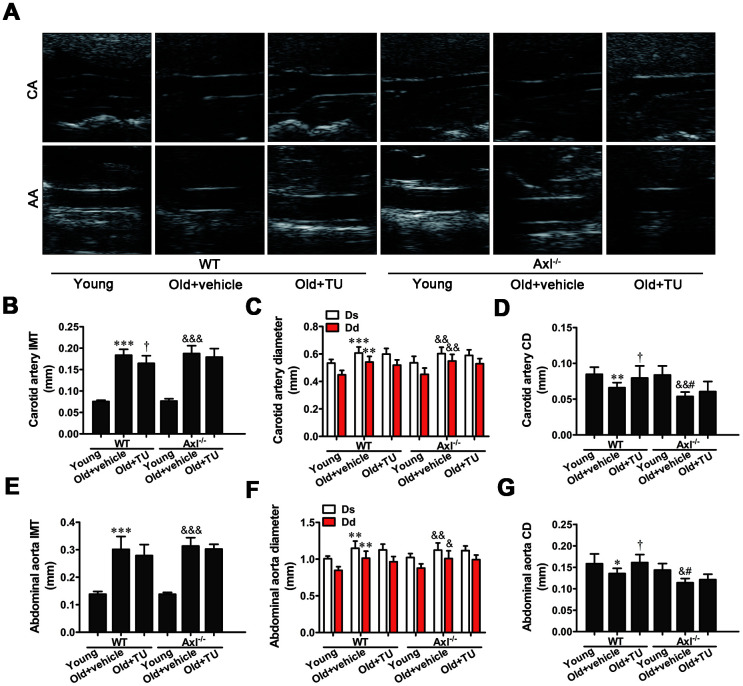
**The role of testosterone and Gas6/Axl in structural changes of aging vessel.** (**A**) Carotid artery (CA) and abdominal aorta (AA) of ultrasonography. (**B**) Intima-media thickness (IMT) of carotid artery. (**C**) Systolic diameter (Ds) and diastolic diameter (Dd) of carotid artery. (**D**) Coefficient distention (CD) of carotid artery. (**E**) IMT of abdominal aorta. (**F**) Ds and Dd of abdominal aorta. (**G**) CD of abdominal aorta. Data are mean ± SD; ^*^P < 0.05, ^**^P < 0.01 and ^***^P < 0.001 *vs.* WT young group; ^†^*P* < 0.05 *vs.* WT old group; ^&^*P* < 0.05, ^&&^*P* < 0.01 and ^&&&^*P* < 0.001 *vs.* Axl^-/-^ young group; ^#^*P*<0.05 *vs.* Axl^-/-^ old group; n = 8 for each group.

Coinciding with prior studies, the intima-media thickness (IMT) was significantly increased in carotid artery and abdominal artery in both WT and Axl^-/-^ old groups compared with WT and Axl^-/-^ young groups (*P*<0.001) (Figure 3A, 3B, 3E). The IMT of carotid artery and abdominal aorta in Axl^-/-^ old group was not significantly thicker than WT old group (Figure 3A, 3B, 3E). With TU treatment, the IMT of carotid artery (*P*<0.05) and abdominal aorta in WT old+TU group decreased compared to WT old group (Figure 3A, 3B, 3E). The IMT of carotid artery and abdominal aorta in Axl^-/-^ old+TU group were not significantly thinner than Axl^-/-^ old group (Figure 3A, 3B, 3E).

Both diastolic diameter (Ds) and systolic diameter (Dd) of the carotid artery and abdominal aorta in WT and Axl^-/-^ old groups were significantly increased compared with WT and Axl^-/-^ young groups (*P*<0.05~*P*<0.001) (Figure 3A, 3C, 3F). However, there was no significant difference of Ds and Dd between Axl^-/-^ old group and WT old group with or without TU treatment (Figure 3A, 3C, 3F). The coefficient distention (CD) of carotid artery and abdominal aorta in WT and Axl^-/-^ old groups were smaller than the WT and Axl^-/-^ young groups (*P*<0.05~*P*<0.01) (Figure 3A, 3D, 3G). The CD of carotid artery (*P*<0.05) and abdominal aorta (*P*<0.05) in Axl^-/-^ old group were smaller than the WT old group (Figure 3A, 3D, 3G). With TU treatment, the CD of carotid artery (*P*<0.05) and abdominal aorta (*P*<0.05) in WT old+TU group were decreased compared with WT old group (Figure 3A, 3D, 3G). There was no significant difference between Axl^-/-^ old+TU group and Axl^-/-^ old group (Figure 3A, 3D, 3G).

### The role of testosterone and Gas6/Axl in vascular stiffness of aging vessel

**Figure 4:**
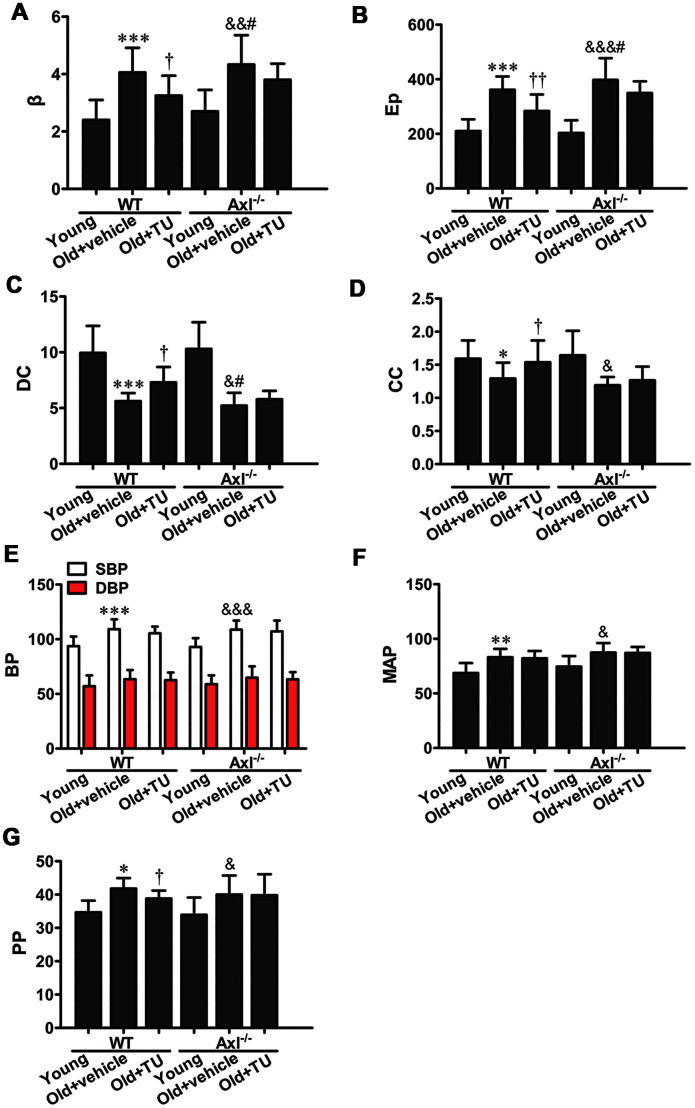
**The role of testosterone and Gas6/Axl in vascular stiffness and blood pressure of aging vessel.** (**A**) Stiffness parameter (β). (**B**) Pressure-strain elastic modulus (Ep). (**C**) Distensibility coefficient (DC). (**D**) Compliance coefficient (CC). (**E**) Systolic pressure (SBP) and diastolic blood pressure (DSP). (**F**) Mean arterial pressure (MAP). (**G**) Pulse pressure (PP). Data are mean ± SD; ^*^P < 0.05, ^**^P < 0.01 and ^***^P < 0.001 *vs.* WT young group; ^†^*P* < 0.05 and ^††^*P* < 0.01 *vs.* WT old group; ^&^*P* < 0.05, ^&&^*P* < 0.01 and ^&&&^*P* < 0.001 *vs.* Axl^-/-^ young group; #*P*<0.05 *vs.* Axl^-/-^ old group; n = 8 for each group.

The stiffness parameter (β) and pressure-strain elastic modulus (Ep) of carotid artery were significantly increased in WT and Axl^-/-^ old groups compared with WT and Axl^-/-^ young groups (*P*<0.01~*P*<0.001) (Figure 4A, 4B). The β (*P*<0.05) and Ep (*P*<0.05) in Ax l^-/-^ old group were higher than in WT old group (Figure 4A, 4B). With TU treatment, the β (*P*<0.05) and Ep (*P*<0.05) of WT old+TU group were significantly decreased compared with WT old group (Figure 4A, 4B). There was no significant difference between Axl^-/-^ old+TU group and Axl^-/-^ old group (Figure 4A, 4B). The distensibility coefficient (DC) and compliance coefficient (CC) of carotid artery were significantly decreased in WT and Axl^-/-^ old groups compared with WT and Axl^-/-^ young groups (*P*<0.05, *P*<0.001) (Figure 4C, 4D). The DC (*P*<0.05) and CC of Axl^-/-^ old group were lower than WT old group (Figure 4C, 4D). With TU treatment, the DC (*P*<0.05) and CC (*P*<0.05) of WT old+TU group were significantly elevated compared with WT old group (Figure 4C, 4D), but no statistical difference was found compared with Axl^-/-^ old+TU group and Axl^-/-^ old group (Figure 4C, 4D).

### The role of testosterone and Gas6/Axl in blood pressure

Both systolic pressure (SBP) and diastolic blood pressure (DBP) were increased in WT and Axl^-/-^ old groups compared with WT and Axl^-/-^ young groups (*P*<0.05~*P*<0.001) (Figure 4E). The SBP and DBP of Axl^-/-^ old group were slightly higher than WT old group (Figure 4E). With TU treatment, the SBP and DBP of WT old+TU group were slightly decreased compared with WT old group (Figure 4E). However, no significant difference was found between Axl^-/-^ old+TU group and Axl^-/-^ old group in SBP and DBP (Figure 4E). The mean blood pressure (MBP) of WT (*P*<0.01) and Axl^-/-^ (*P*<0.05) old groups were increased significantly compared with WT and Axl^-/-^ young groups (Figure 4F). However, there was no significant difference in MBP between WT old+TU group and WT old group, Axl^-/-^ old group and WT old group, Axl^-/-^ old+TU group and Axl^-/-^ old group (Figure 4F).

The pulse pressure (PP) increased in WT (*P*<0.05) and Axl^-/-^ (*P*<0.05) old groups compared with WT and Axl^-/-^ young groups (Figure 4G). PP in Axl^-/-^ group was higher than WT old group (*P*<0.05) (Figure 4G). With TU treatment, the PP in WT old+TU group markedly decreased compared with WT old group (*P*<0.05) (Figure 4G), but no statistical difference was found compared with Axl^-/-^ old+TU group and Axl^-/-^ old group (Figure 4G).

### Testosterone ameliorated vascular fibrosis in aging-related arterial stiffness

**Figure 5:**
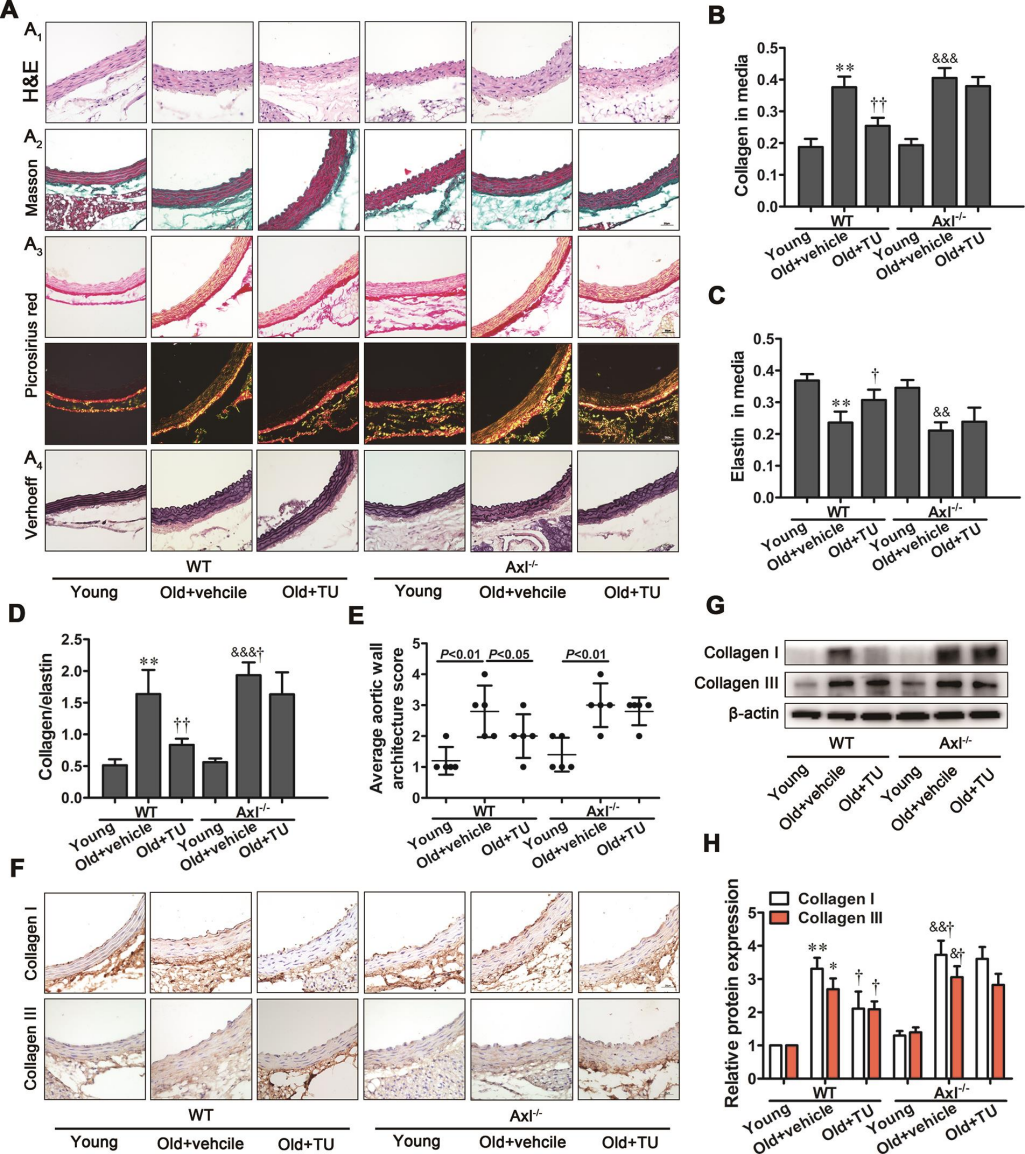
**Testosterone ameliorated vascular fibrosis in aging-related arterial stiffness.** (**A1**-**4**) Representative H&E staining, Masson trichrome staining, Picrosirius red staining and Verhoeff Van Gieson (scale bar: 20μm). (**B**–**E**) Quantitative analyses of collagen content, elastin content, collagen content/elastin content and average aortic wall architecture score. (**F**) Immunohistochemical staining for collagen I and III (brown staining considered positive staining; scale bar: 20μm). (**G**) Representative Western blots of collagen I and III. (**H**) Western blot analyses of collagen I and III. Data are mean ± SD; ^*^P < 0.05 and ^**^P < 0.01 *vs.* WT young group; ^†^*P* < 0.05 and ^††^*P* < 0.01 *vs.* WT old group; ^&^*P* < 0.05, ^&&^*P* < 0.01 and ^&&&^*P* < 0.001 *vs.* Axl^-/-^ young group; ^#^*P*<0.05 *vs.* Axl^-/-^ old group; A-F: n = 5 for each group, G-H: n = 3 for each group.

The Masson trichrome staining, Picrosirius red staining and Verhoeff’s Van Gieson staining results showed vascular fibrosis in the media. The collagen contents, collagen-to-elastin ratio, average aortic wall architecture score significantly increased, and elastin content decreased in WT and Axl^-/-^ old groups compared with WT and Axl^-/-^ young groups (*P*<0.01~*P*<0.001) (Figure 5A_2~4_, 5B–5E). And collagen-to-elastin ratio (*P*<0.05) of Axl^-/-^ old group were higher than WT old group (Figure 5A_2~4_, 5B, 5D, 5E). With TU treatment, the collagen content (*P*<0.01), collagen-to-elastin ratio (*P*<0.01) and average aortic wall architecture score (*P*<0.05) markedly decreased, and elastin content (*P*<0.05) increased in WT old+TU group compared to WT old group (Figure 5A_2~4_, 5B, 5D, 5E). But there was no significantly difference in collagen content, collagen-to-elastin ratio, average aortic wall architecture score and elastin content between Axl^-/-^ old group and Axl^-/-^ old+TU group (Figure 5A_2~4_, 5B, 5D, 5E).

The immunohistochemical staining and western blot results showed that collagen I and III expression were significantly increased in WT and Axl^-/-^ old groups compared with WT and Axl^-/-^ young groups (*P*<0.05~*P*<0.01) (Figure 5F, 5G, 5H). The collagen I and III expression in Axl^-/-^ old group were higher than WT old group (Figure 5F, 5G, 5H), and were significantly decreased in WT old+TU group compared to WT old group (*P*<0.05) (Figure 5F, 5G, 5H). However, between the Axl^-/-^ old+TU group and Axl^-/-^ old group, no statistical difference was found in collagen I and III (Figure 5F, 5G, 5H).

### The protective effects of testosterone on vascular aging was attributed to inhibition of MMPs expression and activity

**Figure 6:**
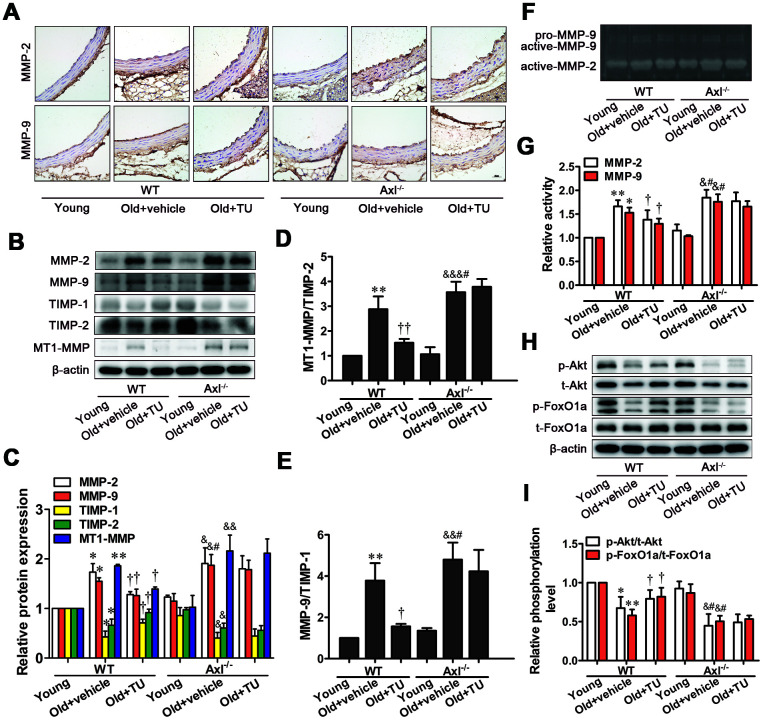
**The protective effects of testosterone on vascular aging were attributed to inhibition of MMPs expression and activity through promotion of Akt/FoxO1a phosphorylation.** (**A**) Immunohistochemical staining for MMP-2 and MMP-9 (brown staining considered positive staining; scale bar: 20μm). (**B**) Representative Western blot of MMP-2, MMP-9, TIMP-1, TIMP-2 and MT1-MMP. (**C**–**E**) Western blot analyses of MMP-2, MMP-9, TIMP-1, TIMP-2, MT1-MMP, MT1-MMP/TIMP-2 and MMP-9/TIMP-1. (**F**) Representative Gelatin Zymography of MMP-2 and MMP-9 activity. (**G**) Gelatin Zymogrphy analyses of MMP-2 and MMP-9 activity. (**H**) Representative Western blot of p-Akt, t-Akt, p-FoxO1a and t-FoxO1a. (**I**) Western blot analyses of p-Akt, t-Akt, p-FoxO1a and t-FoxO1a. Data are mean ± SD; ^*^P < 0.05 and ^**^P < 0.01 *vs.* WT young group; ^†^*P* < 0.05 and ^††^*P* < 0.01 *vs.* WT old group; ^&^*P* < 0.05, ^&&^*P* < 0.01 and ^&&&^*P* < 0.001 *vs.* Axl^-/-^ young group; ^#^*P*<0.05 *vs.* Axl^-/-^ old group; A: n = 5 for each group, B-H: n = 3 for each group.

Immunohistochemical staining, Western blot and Zymography results showed that MMP-2 and MMP-9 expression and activity were significantly increased in WT and Axl^-/-^ old groups compared with WT and Axl^-/-^ young groups (*P*<0.05, *P*<0.01) (Figure 6A–6C, 6F, 6G). The MMP-2 and MMP-9 expression and activity in Axl^-/-^ old group were higher than WT old group (*P*<0.05) (Figure 6A–6C, 6F, 6G), and were significantly decreased in WT old+TU group compared with WT old group (*P*<0.05) (Figure 6A–6C, 6F, 6G). But there was no significant difference between the Axl^-/-^ old group and Axl^-/-^ old+TU group (Figure 6A–6C, 6F, 6G).

The MT1-MMP expression, MT1-MMP to TIMP-2 ratio and MMP-9 to TIMP-1 ratio were significantly elevated, while TIMP-1 and TIMP-2 expressions were decreased in WT and Axl^-/-^ old groups compared with WT and Axl^-/-^ young groups (*P*<0.05~*P*<0.001) (Figure 6B–6E). The MT1-MMP expression, MT1-MMP to TIMP-2 ratio (*P*<0.05) and MMP-9 to TIMP-1 ratio (*P*<0.05) in Axl^-/-^ old group were higher than WT old group (Figure 6B–6E), and were significantly decreased in WT old+TU group compared to WT old group (*P*<0.05~*P*<0.01) (Figure 6B–6E). There was not significantly different between the Axl^-/-^ old+TU group and Axl^-/-^ old group (Figure 6B–6E).

### Elevated phosphorylation of Akt/FoxO1a was implicated in the protective effects of testosterone on vascular aging

The phosphorylation levels of Akt and FoxO1a were significantly decreased in WT and Axl^-/-^ old groups compared with WT and Axl^-/-^ young groups (*P*<0.05~*P*<0.01) (Figure 6H, 6I). The phosphorylation levels of Akt (*P*<0.05) and FoxO1a (*P*<0.05) in Axl^-/-^ old group were lower than WT old group (Figure 6H, 6I). With TU treatment, the phosphorylation levels of Akt (*P*<0.05) and FoxO1a (*P*<0.05) were significantly increased in WT old+TU group compared with WT old group (Figure 6H, 6I). But there was no significant difference in phosphorylation levels of Akt and FoxO1a between the Axl^-/-^ old+TU group and Axl^-/-^ old group (Figure 6H, 6I).

## DISCUSSION

In this study, we have further proved the protective effect of testosterone on vascular aging and the role of Gas6/Axl pathway in this process. Firstly, our findings revealed that testosterone alleviated cell senescence and age-related vascular remodeling through Gas6/Axl pathway. Furthermore, in this study we show that Akt/FoxO1a pathway was involved in the protective effect of testosterone on vascular aging. These results suggest that testosterone plays a pivotal role in vascular aging, and testosterone replacement therapy (TRT) may be useful to treat age-related vascular disease.

Vascular aging is considered as an important trigger promoting age-related cardiovascular diseases [24]. Aging vessel is associated with increased deposition of collagen and breakages in the elastic laminae. The overall effects of aging on vessel result in increased arterial stiffness and inflammation, thicker walls and lumen dilation, and a diminished ability to contract and relax. In this study, we also observed that IMT, Ds, Dd, β, Ep, collagen contents, collagen-to-elastin ratio and average aortic wall architecture score of aorta were increased in the old mice. Furthermore, the elevated density of collagen I, collagen III, as well as the activity of MMPs suggested the increased collagen deposition in old mice. These observations suggested that evident vascular aging existed in aging mice.

Testosterone, the main sex hormone, plays a crucial role to maintain male physiological function, and the levels undergo age-related decline. Testosterone deficiency has been shown to be associated with increased carotid-femoral PWV, arterial stiffness, microvascular dysfunction, and emerged as an important predictor of future cardiovascular events both in the general population and in patients with disease states [25, 26]. Data regarding the effects of testosterone replacement on cardiovascular events are contradictory. A recent retrospective study reported patients who had a normal testosterone level after TRT had lower all-cause mortality, MI, and stroke [27]. Consistent with previous studies, we found the serum level of testosterone decreased in aging mice. Compared with control group, the aging mice with testosterone treatment were in a better condition and more active. Decreased IMT, luminal dilation, vascular stiffening, collagen content, collagen-to-elastin ratio, and average aortic wall architecture score of aorta were observed in aging mice with testosterone treatment. Meanwhile, density of collagen I and collagen III, and activity of MMPs were reduced. Therefore, testosterone could improve age-related vascular remodeling, which demonstrates TRT can delay vascular aging.

The Gas6/Axl pathway has been implicated in cell survival and proliferation, cell adhesion and migration, homeostasis, and inflammatory cytokine release. The Gas6/Axl pathway has also been implicated in in the development of vascular calcification, vascular remodeling and atherosclerosis [14–16]. Gas6 was inversely associated with age, and positively correlated with testosterone level [28]. Testosterone might directly activate androgen-response elements of the Gas6 gene to influence Gas6 gene expression and protein production [20, 29]. However, whether Gas6/Axl is involved in the process of age-related vascular remodeling improved by testosterone is unknown. Our previous study showed that Gas6/Axl played an important role in testosterone alleviating angiotensin II-induced VSMC senescence [23]. To further prove the anti-aging effect of testosterone *in vivo*, we established Axl^-/-^ aging mice model by natural aging, and then gave testosterone treatment. In accord with previous study, we found the levels of Gas6 and Axl decreased in aging mice. Testosterone replacement could increase the expression of Gas6, but Axl expression was still lower in the Axl^-/-^ aging mice. More importantly, vascular stiffness, collagen content, collagen-to-elastin ratio, average aortic wall architecture score of aorta, and activity of MMPs were significantly increased in the Axl^-/-^ aging mice, but no statistical difference was found compared with testosterone-treated Axl^-/-^ aging mice. This data indicated that knocking out Axl aggravated age-related vascular remodeling and counteracted the protective effect of testosterone on vascular remodeling. However, we noticed that there was no statistical difference in IMT and diameter of aorta between Axl^-/-^ aging mice and WT aging mice. Gsa6/Axl has been implicated in cell survival, proliferation, migration, adhesion and decreased apoptosis [30]. Hence, we hypothesized that, when Axl was knocking out, Gas6-induced cell proliferation might be attenuated, apoptosis increased, and cells of aorta were induced, which influenced vascular thickness.

Cellular senescence was considered to play an important role in vascular aging [9, 10]. Cellular senescence can cause disorders of ECM metabolism, initiate vascular remodeling, and finally result in vascular stiffness [31, 32]. To explore the role of testosterone on cellular senescence, we detected telomere length, the expression of p16^INK4a^ and p21^Cip1^ and the β-galactosidase staining. We found telomere length was short, p16^INK4a^ and p21^Cip1^ expression and SA-β-Gal staining rate were elevated in the aortas of aging mice. The above performance suggested that cell senescence was existed in vascular aging. With testosterone treatment, the aging features of cell senescence were markedly alleviated, suggesting testosterone could improve cell senescence in aging vessel. Axl knockout animals exhibited enhanced aging performance and showed the blunted anti-senescence effect of testosterone. Together, these data suggests that Gas6/Axl pathway has been involved in the anti-senescence effect of testosterone in vivo.

Age-associated increase in arterial pressure is a clinical hallmark of vascular stiffness. In older individuals, the age-dependent change in blood pressure is determined to a greater extent by central conduit vessel stiffness, which leads to an increase in systolic pressure and declines in diastolic pressure. Isolated systolic hypertension emerges as the most common form of hypertension in older adults. Increased systolic pressure and pulse pressure can profoundly influence blood vessel and heart biology. Thus, isolated systolic hypertension and pulse pressure, as the clinical surrogate markers of vascular stiffness, even when mild in severity, is associated with an appreciable increase in cardiovascular disease risk [33]. Low testosterone level was positively related to hypertension [34]. We found that SBP and PP were increased in aging mice, and testosterone treatment improved these changes. However, SBP and PP were not significantly different in Axl ^-/-^ aging mice compared to WT aging mice with or without testosterone treatment. Korshunov VA [35] and Batchu N [36] found Axl deficiency could reduce SBP level in deoxycorticosterone acetate (DOCA)-salt hypertension model, and gas6/Axl contributes to vascular endothelial dysfunction, and remodeling via inhibiting vascular apoptosis, in the late phase of DOCA-salt hypertension. The role of Gas6/Axl on hypertension needs further investigation.

Gas6/Axl has also been reported to mediate survival signals through activation of Akt. As a downstream effector of the Akt, FoxO can be phosphorylated by p-Akt [37]. Our previous study showed that Akt/FoxO signaling pathway was involved in cell cycle regulation, MMPs metabolism, and the anti-senescence effect of testosterone on VSMC senescence [19, 23]. In this study, we found that the phosphorylation of Akt and FoxO1a decreased in aging aortas, and testosterone could restore the phosphorylation of Akt and FoxO1a. Knocking out Axl suppressed the phosphorylation of Akt and FoxO1a. Therefore, we conclude that Gas6/Axl take part in the anti-vascular aging effect of testosterone, which is most likely achieved through the activation of the Akt and FoxO1a signaling pathway.

In conclusion, Gas6/Axl plays a pivotal role in testosterone alleviating vascular aging through modulating Akt/FoxO1a pathway. The present study reveals a molecular mechanism underlying the protective effects of testosterone on vascular aging, and provides a theoretical basis for TRT. The anti-vascular aging effects of testosterone might contribute to its improving effect on age-related cardiovascular diseases.

## MATERIALS AND METHODS

### Animal model and drug treatment *in vivo*

Ninety Axl^-/-^ male mice were offered by Professor Pan J. from Shandong Normal University (Jinan, China). Ninety wild-type male mice were purchased from Beijing Vital River Laboratory Animal Technology Co. Ltd. (Beijing, China), all mouse lines were on a B6129SF2/J genetic background. The animals were housed at 22°C with 12-h light-dark cycles and maintained on a normal chow diet and water. Mice of two gene types were then randomized into three groups: the young group (n = 30, 3 months old), the aging group (n = 30, 18 months old) and the testosterone undecanoate treatment group (TU, n = 30, 18 months old). Mice in TU group were given testosterone undecanoate (37.9 mg/Kg) by subcutaneous injection on the back at fifteen-month-old, once a month, a total of three times. Old group received solvent reagent (corn oil) by the same method. At the end of the experiment, the mice were sacrificed. All animals procedures were performed corresponding to the institutional guidelines of Qilu Hospital of Shandong University and were approved by Shandong University Institutional Animal Care and Use Committee.

### Ultrasonography

Ultrasonography was performed at 3, 15 and 18 month. All animals were anesthetized and laid supine on a heated table, and warmed ultrasound transmission gel was placed on the chest. The two-dimensional imaging was performed by using the Real-TimeMicro Visualization Scanhead (RMV 704) with a central frequency of 40 MHz at the mechanical transducer (Vevo 770; VisualSonics, Toronto, ON, Canada). Intima-media thickness (IMT), diastolic diameter (Dd) and systolic diameter (Ds) measurements were performed according to previously validated protocols in humans. Two-dimensional ultrasonography was used to calculate arterial stiffness index (β), Peterson’s elastic modulus (Ep), distensibility coefficient (DC) and compliance coefficient (CC). All measurements reported represented the means of three consecutive cardiac cycles and were performed by the same investigator.

β, Ep, DC and CC were estimated automatically by the following formulae:

β = ln {(Ps / Pd) / [(Ds – Dd) / Dd]}

Ep = (ΔP / ΔD) × Dd = [(Ps – Pd) / (Ds – Dd)] × Dd (10^6^dyn / cm^2^)

DC = (2ΔD / D) / ΔP

CC = (πD × ΔD) / 2ΔP

### Blood pressure measurement

Systolic blood pressure (SBP), diastolic blood pressure (DBP), mean blood pressure (MBP) and heart rate were measured by a pulse based tail-cuff method with a photoelectric device (Natsume, Tokyo, Japan) at 3, 15 and 18 month. Blood pressure and heart rate were reported as a mean of 3 consecutive measurements.

### Tissue preparation

The aorta was rapidly removed and washed in a phosphate-buffered solution (PBS). The upper part of the aorta (approximate 5 mm) was fixed in 4% neutral formaldehyde for 48 h. The tissues were dehydrated in ethanol, embedded in paraffin, and cross sectioned (4-μm thickness) for histology staining. The remaining portions of the aortas were immediately frozen in liquid nitrogen and stored at -80 °C for molecular studies.

### Western blotting analysis

The aortas were snap-frozen in liquid nitrogen, pulverized, and resuspended in ice-cold lysis buffer (Beyotime). Lysates were placed on ice for 30 min to solubilize, and supernatant was collected by centrifugation (15,000g) for 15 min at 4°C. Protein concentrations were determined with the Bicinchoninic Acid Assay (BCA) method, and then the supernatants were analyzed by SDS-polyacrylamide gel electrophoresis. About 30 μg of total protein per well was loaded and then electrophoresed at 80 V for 30 minutes and 110V for 60 minutes. The gels were then transferred at 200mA for 120minutes onto a PVDF membrane. Membranes were blocked with 5% non-fat milk in TBS with 0.1% Tween-20 for 1.5 hours. After blocking, membranes were incubated at 4°C for overnight with the specific primary antibodies in antibody diluent (P0023A, Beyotime). Gas6 (Goat, Santa Cruz Biotechology, USA), Axl (Rabbit, Cell Signaling Technology, USA), p16^INK4a^ (Mouse, Santa Cruz Biotecholog, USA), p21^Cip1^ (Rabbit, Santa Cruz Biotecholog, USA), collagen I (Rabbit, AbcamAbcam, USA), collage III (Rabbit, Abcam, USA), MMP-2 (Rabbit, Abcam, USA), MMP-9 (Rabbit, Abcam, USA), TIMP-1 (Rabbit, Santa Cruz Biotecholog, USA), TIMP-2 (Rabbit, Santa Cruz Biotecholog, USA), MT1-MMP (Rabbit, Santa Cruz Biotecholog, USA), Akt (Rabbit, Cell Signaling Technology, USA), p-Akt (Rabbit, Cell Signaling Technology, USA), FoxO1a (Rabbit, Cell Signaling Technology, USA), p-FoxO1a (Rabbit, Cell Signaling Technology, USA) and β-actin (Mouse, Zhongshan Biotech, China), followed by anti-IgG horseradish peroxidase-conjugated secondary antibody. The membrane bands were visualized by use of chemiluminescence (Millipore) and quantified by densitometry with application of the Image J software (U.S. National Institutes of Health, Bethesda, USA).

### Quantitative real-time RT-PCR

Total RNA was extracted using TRIzol reagent (Takara), then quantified by spectrophotometry and reverse-transcribed with the Reverse Transcriptase System (TAKARA) and oligo (dT) primers. RT-PCR was performed using the following primers: β-actin, forward 5’-CAT GTA CGT TGC TAT CCA GGC-3’and reverse 5’-CTC CTT AAT GTC ACG CAC GAT-3’; Gas6, forward 5’-GCT TCT GCT GCT CCT GCT G-3’and reverse 5’-ATA GTC CGT CTC GGG GTC GT-3’; Axl, forward 5’-TGA TAA CAC CCA GAC CCA GG-3’ and reverse 5’-TGA CTC CCT TGG CAT TGT GG-3. The mRNA levels for Gas6 and Axl were determined by a real-time PCR thermocycler (CFX Real-Time PCR cycler; Bio-Rad) with SYBR green as fluorescence dye. All values obtained were normalized to mouse β-actin.

### Telomere length measurement

The genomic DNA of the descending aorta was extracted and the CT values of telomere and single copy gene were measured by RT-PCR. The data was collected by Bio-Rad iQ5 system. Relative ratio of telomere/single copy gene = 2^-ΔΔCT^. The primer sequences were as follows: Tel 1 5’-GGT TTT TGA GGG TGA GGG TGA GGG TGA GGG TGA GGG T-3’, Tel 2 5’-TCC CGA CTA TCC CTA TCC CTA TCC CTA TCC CTA TCC CTA-3’; HBG 1 5’-GCT TCT GAC ACA ACT GTG TTC ACT AGC-3’, HBG 2 5’-CAC CAA CTT CAT CCA CGT TCA CC-3’.

### Senescence-associated β-galactosidase staining

After the β-galactosidase staining solution was added, the sections of aortic walls were incubated at 37 °C without CO2 overnight. Cells which had blue-green granules in their cytoplasm were regarded as positive staining. For each group, six fields of the sections were selected randomly to calculate the positive staining rate using Image-Pro Plus 6.0 software.

### ELISA

The serum level of Gas6 (R&D Systems, USA) and testosterone (Abcam, USA) was measured by ELISA assay according to the manufacturer’s instruction.

### Histopathological analysis

Morphological changes of aortic walls were observed in sections stained with Hematoxylin-eosin staining. The collagen deposition of aortic walls was evaluated with Sirius red staining and Masson staining. Elastin content was measured by Verhoeff staining. The relative positive staining area for collagen and elastin were measured by an image analysis system (Image-Pro Plus, Version 6.0; Media Cybernetics, Houston, TX).

### Immunohistochemical analysis

Sections were conducted antigen retrieval with a microwave-based method. Primary antibodies for collagen I (Rabbit, Abcam, USA), collagen III (Rabbit, Abcam, China), MMP-2 (Rabbit, Abcam, USA), MMP-9 (Rabbit, Abcam, USA) were applied and incubated overnight in PBS at 4°C. After three washes in PBS, (10min/wash), sections were incubated with the appropriate secondary antibody for 30 minutes at 37°C. The stained sections were developed with diaminobenzidine and counterstained with hematoxylin. The results were viewed under a confocal FV 1000 SPD laser scanning microscope (Olympus, Japan) and evaluated using Image-Pro Plus software.

### Zymography

MMP activities of the aortic segment were assessed with zymography. Briefly, samples were minced and homogenized in ice-cold PBS, then tissues were centrifuged to collect proteins. Protein content was measured by a Bio-Rad protein assay, and SDS-polyacrylamide gel electrophoresis zymography was performed. One part of homogenate containing 30 μg of protein was loaded on a 10% SDS-polyacrylamide gel containing 0.1% gelatin.

### Statistical analyses

All data was presented as mean ± SD. SPSS 20.0 (SPSS, Chicago, IL) was used for statistical analysis. Results were compared by one-way ANOVA, followed by Tukey-Kramer post hoc test and independent samples *t* test. *P*<0.05 was considered statistically significant.
